# Editorial Notes

**Published:** 1895-02

**Authors:** 


### Editorial Notes.

DRS. C. D. Hurt and E. C. Davis have removed to 545 Equitable building.

---

DR. H. P. MCCANSLAND, representing Parke, Davis & Co., called on the RECORD last week.

---

THE FIRST meeting of the Board of Medical Examiners was held a few days ago. The dates for the examination of applicants will be determined later. The officers of the board are: Dr. A. A. Smith, Hawkinsville, President; Dr. W. O'Daniel, Bullards, Vice-President; and Dr. Frank M. Ridley, LaGrange, Secretary and Treasurer.

DR. ALFRED F. Harrington, formerly of West Point, has recently located here, and is associated with Dr. Wm. Perrin Nicolson. Dr. Harrington graduated at the Atlanta Medical College, Class of '92, and among his many friends none wish him greater success than his classmates.

---

THE OFFICIAL acceptance of Hondura s has just been received by President Collier of the Cotton States and International Exposition. President Bonilla writes that frequent revolutions have retarded the industrial progress of Honduras, but the best possibleexhibit will be made.

---

MESSRS. JOHN Sprague, "Jack" Pryor and Wm. W. Curtis, representing Sharp & Dohme, Baltimore, are in the city at this writing, and more affable, polished and hustling "gentlemen of the road" couldnot befound in a day's journey. They report fine business, and deserve it, as the quality of their goods and the character of the firm and representatives are all and more than is claimed for them by their many patrons and friends.

---

WE REGRET to chronicle the continued illness of Dr. Dan H. Howell, business manager of the RECORD. He desires to return thanks to the many friends for their kind expressions of sympathy and wishes for his speedy recovery. In this connection, the managing editor desires to say to the business patrons of the RECORD, that delayed correspondence, as the result of Dr. Howell's indisposition, will be attended to at once, under the direction of the business manager.

---

IN THE January issue of the RECORD, the papers by Dr. J. M. Mathews, of Louisville, and Dr. A. Lapthorn Smith, of Toronto, appeared under the head of "Original Communications." They should have been credited to *Mathews' Medical Quarterly*, and this error is due to the oversight of the printer. One or two other articles appeared without proper credit being given. We can assure our brethren of the medical press that we value as highly as anyone that unwritten law of journalistic ethics—to do justice, and compliment the man or journal for originality and ability.

#### GONORRHŒA.

℞. Liquoris Potassæ .......f ʒ j.
Balsami Copaibæ ......f ℥ ss.
Tincturæ Cubebæ ......f ʒ vj.
Liquoris Morphinæ Sulphatis ..................f ℥ j.
Aquæ Camphoræ, q. s. ad f ℥ vj.
M. Sig.—Take a tablespoonful four times a day.
—*D. Hayes Agnew*.

#### HEADACHE.

℞. Ætheris ................
Spiritus Ammoniæ aromatici ..............aā f ʒ .
Aquæ Camphore ......f ʒ x.
Tincturæ Cardamomi compositæ ............f ʒ j.
Misce pro hausto.
Sig.—Take two to three times a day. (*In nervous headache.*)
—*Brande*.

#### EPISTAXIS.

℞. Strychnæ, Sulphatis .....gr. ¼
Tincturæ Ferri Chloridi. f ʒ ij.
Vini Ergotæ ........ ...f ℥ ss.
Elixiris simplicis..... f ℥ iss.
Aquæ destillatæ, q. s. ad f ℥ vj.
M. Sig.—Take a tablespoonful three times a day. (*In anæmic cases.*)
—*Lombe Athill.*

#### PARALYSIS.

℞. Strychninæ Sulphatis gr. j.
Acidi Arseniosi.......gr. ij.
Extracti Belladonnæ..gr. v.
Quininæ Sulphatis....
Pilulæ Ferri Carbonatis aā ℈ ij.
Extracti Taraxaci.....℈ j.
Misce et fiant pilulæ No. xi.
Sig.—Take one pill three times a day. (*In paralysis agitans of aged people.*)
—*S. W. Gross.*

**REDUCED PRICES.**

**MELLIER'S "ELLIOTT" SADDLE-BAGS.**

*Black or Russet Leather.*

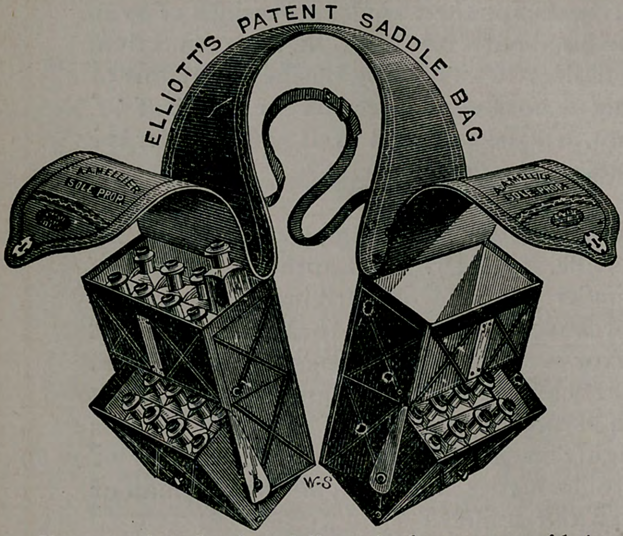

**Small, - - $6.00**
Eight ¾-oz. vials, sixteen 1½-oz. vials.

**Large, - - $7.00**
Ten ¾-oz. vials, twenty 1½-oz. vials.

**Extra Large, - $8.00**
Twelve 1½-oz. vials, sixteen 3-oz. vials.

**Upon receipt of price, delivered, charges prepaid, to your nearest Express Office.**

**MELLIER DRUG COMPANY,** Sole Proprietors,

*2112 Lucas Place and 721 Locust St., ST. LOUIS.*

Please mention Southern Medical Record.

### HEMORRHAGE.

℞. Ammonii Carbonatis, ʒ ij.
Tincturæ Opii deodoratæ . . . . . . . . . . . . . . f ℥ iss. (!)
Extracti Glycyrrhizæ fluidi . . . . . . . . . . f ʒ vj.
Aquæ destillatæ, q. s. ad f ℥ vj.

M. Sig.—Take a tablespoonful every two hours. (*After hemorrhage ad deliquium.*)
—*Carson.*

### INSOMNIA.

℞. Antimonii et Potassi Tartratis . . . . . . . . . . . . . . gr. j–ij.
Morphinæ Sulphatis . . gr. iss.
Aquæ Laurocerasi . . . . f ℥ j.

M. Sig.—A teaspoonful every two, three, or four hours. (*In the delirium and wakefulness of fevers.*)
—*Bartholow.*

### HICCOUGH.

℞. Hydrargyri Chloridi mitis, gr. j

Sacchari Lactis . . . . . . . . . . . ʒ ss.

Misce et fiant chartulæ No. xij.

Sig.—Take one powder every hour. (*In obstinate cases with extreme debility.*)
—*Gerhard.*

### INGROWING TOE-NAIL.

℞. Liquoris Potassæ . . . . . f ʒ ij.

Aquæ destillatæ . . . . . . . f ℥ j.

M. Sig.—Apply with pledgets of cotton-wool.
—*Norton*

### FROST-BITE.

℞. Iodi . . . . . . . . . . . . . . . . . . . . . . . ℈ j.
Potassii Iodidi . . . . . . . . . . gr. iv.
Aquæ destillatæ . . . . . . . . *M* vi.
Adipis . . . . . . . . . . . . . . . . . . ℥ j.

M. Apply daily. (*With unbroken skin.*)
—*Hebra.*

DIVERS STATUTES FOR PHARMACISTS.—The *British and Colonial Druggist* has been making a study of the pharmacy laws prevailing in the different States and Territories, and publishes the following summary:

In the following all must undergo the State's examination: Washington, Mississippi, Nebraska, Ohio, Illinois, Rhode Island, Massachusetts, Michigan, Minnesota, New York, Wisconsin, Arkansas, Maine, New Jersey, Pennsylvania, Utah, West Virginia.

The following accept graduates of regular colleges of pharmacy: Florida, District of Columbia, Iowa, Missouri, Oklahoma.

Georgia.—This State accepts the certificates of Germany, France, Great Britain, Ireland and Ontario. All others must submit to examination, but are allowed to practice until the regular meeting of the Board, provided they hold a certificate.

Virginia accepts the certificates of Great Britain and Ireland, but a bill is before the State Legislature which will render examination compulsory.

---

ANATOMICAL ACCIDENTS.—He kissed her passionately upon her reappearance.—*Jefferson Souvenir.*

She whipped him upon his return.—*Burlington Hawkeye.*

He kissed her back.—*Atlanta Constitution.*

We thought she sat down upon her being asked.—*Saturday Gossip.*

She fainted upon his departure.—*Lynn Union.*

He kicked the tramp upon his sitting down.—*American Pharmacist.*

We feel compelled to refer to the poor woman who was shot in the oil regions.—*Medical World.*

And why not drop a tear for the man who was stabbed in the rotunda and for him who was kicked on the highway.—*Medical Age.*

Why not mention the fact of the man being shot in the water-works.—*Cal. Med. Jour.*

How about the woman who was hurt in the fracas?—*Railway Age.*

Page(s) missing

